# Addressing Challenges with the Categorization of Foods Processed at Home: A Pilot Methodology to Inform Consumer-Facing Guidance

**DOI:** 10.3390/nu12082373

**Published:** 2020-08-08

**Authors:** Rachel Bleiweiss-Sande, Caitlin P. Bailey, Jennifer Sacheck, Jeanne P. Goldberg

**Affiliations:** 1 Friedman School of Nutrition Science & Policy, Tufts University, Boston, MA 02111, USA; jeanne.goldberg@tufts.edu; 2 Milken Institute School of Public Health, The George Washington University, Washington, DC 20052, USA; caitlin.bailey@tufts.edu (C.P.B.); jsacheck25@email.gwu.edu (J.S.)

**Keywords:** dietary guidance, food processing, food classification systems, processing, ultra-processed, NOVA, home cooking, homemade foods, home-prepared foods

## Abstract

The objective of this study was to inform consumer-facing dietary guidance by (1) adapting the current University of North Carolina at Chapel Hill (UNC) food processing framework to include a home processing (HP) component and (2) pilot testing the adapted version using a nationally representative sample of foods consumed in the U.S. The UNC framework was adapted to include guidelines for categorizing home-prepared (HP) foods. The original UNC and adapted HP frameworks were used to code dietary recalls from a random sample of National Health and Nutrition Examination Survey (2015–2016 cycle) participants (*n* = 100; ages 2–80 years). Percent changes between the UNC and HP adapted frameworks for each processing category were calculated using Microsoft Excel, version 16.23. Participants were 56% female, 35% non-Hispanic white (mean age = 31.3 ± 23.8). There were 1,376 foods with 651 unique foods reported. Using the HP compared to the UNC framework, unprocessed/minimally processed foods declined by 11.7% (UNC: 31.0% vs. HP: 27.4%); basic processed foods increased by 116.8% (UNC: 8.2% vs. HP: 17.8%); moderately processed foods increased by 16.3% (UNC: 14.2% vs. HP: 16.6%); and highly processed foods decreased by 17.8% (UNC: 46.5% vs. HP: 38.2%). Home-prepared foods should be considered as distinct from industrially produced foods when coding dietary data by processing category. This has implications for consumer-facing dietary guidance that incorporates processing level as an indicator of diet quality.

## 1. Introduction

Various forms of food processing such as curing, drying and fermenting have been used by humans for over two million years [1]. Over the course of the 20th century, rapid advances in processing technology combined with prevailing economic and social conditions have led to drastic changes in food production at an industrial scale [1,2]. Despite this broad historical context, food processing as an issue of concern with respect to food quality and its effects on health have only recently begun to occupy center stage among researchers.

It is widely accepted that poor diet, as a key component of the energy balance equation, is an important target for obesity and chronic disease prevention, particularly among individuals from socio-economically disadvantaged backgrounds [3]. However, communicating evidence-based actionable messages about healthful eating remains a challenge [4]. Within this context, decreasing intake of processed food, and in particular the most highly or “ultra-processed” products, has emerged as a potential target for obesity and chronic disease prevention [5,6]. 

Current processing research is complicated by the fact that there is no commonly agreed upon approach to categorize foods based on the extent of processing [6,7]. Consistent definitions for categorizing foods according to extent of processing are needed to both describe the possible role of processed food consumption in disease and to incorporate information about processing into health communication efforts. A number of frameworks have been proposed by organizations globally. These include the European Prospective Investigation into Cancer (EPIC) in Europe [8], the International Food Information Council (IFIC) in the U.S. [9], the International Food Policy Research Institute (IFPRI) in Guatemala [10], the Mexican National Institute of Public Health (MNIPH) [11] and the NOVA system in Brazil [12].

In the U.S., researchers at the University of North Carolina at Chapel Hill (UNC) adapted a version of the NOVA framework to capture the complexity of the U.S. food supply [12,13]. The UNC framework incorporates expanded processing categories (Table 1). Several studies have demonstrated that the UNC system has the highest inter-rater reliability compared to other classification systems using European [14] and U.S. food samples [15]. As described by the authors of NOVA, the systems were developed to “group foodstuffs according to the extent and purpose of the industrial processing applied to them [12]”. In the case of the UNC system, industrial processing was operationalized to include “commercial manufacturing operations that convert raw agricultural commodities into packaged, canned, frozen, dried, fermented, formulated, and otherwise modified forms of food but excluding processing (i.e., cooking) by the food service industry [13]”. Therefore, the UNC system provides processing categorization for packaged foods available from food vendors in the U.S., but excludes any product without a barcode (such as certain agricultural products and any mixed dish prepared on a non-industrial scale) [13].

While the UNC and other systems have contributed substantially to the global conversation on highly processed food products, issues arise when applying these frameworks to foods processed in the home. Research indicates that the percent of Americans who cook at home has increased between 2003 and 2016, with just over 50% reporting home cooking activities in 2016 [16]. Of the food processing classification systems included in a review of five frameworks [7], only EPIC [17] and the MNIPH [11] systems explicitly reference home-prepared foods. Of these, only the MNIPH system acknowledges that certain home-prepared foods are nearly impossible to systematically disaggregate [11]. In contrast, systems such as NOVA and EPIC have proposed that mixed dishes without product specifications or barcodes (e.g., home-prepared foods) be coded as their ingredient parts [17,18]. While coding foods based on their ingredients may lead to greater precision, this strategy can only be used when ingredient data are available; missing ingredient data defaults the user to code foods as their store-bought equivalent.

The NOVA and other processing classification systems have received extensive criticism from the scientific community regarding their consistency [19], reliability [14,15], validity [14,15], and policy implications [19,20]. Importantly, misclassification of home-prepared foods as their store-bought equivalent may lead to inappropriate associations of home-cooked items with less desirable health outcomes or misrepresentation of nutrient profiles associated with highly processed food consumption. Despite these criticisms, little has been done to attempt to improve on their usability. A more rigorous conceptualization of processed food classification should include workable directions for categorizing home-prepared foods as distinct from industrially produced foods, even when comprehensive ingredient data is unavailable. Therefore, our objective was to adapt the current UNC food processing framework to include a home processing (HP) component to more accurately describe the processing categories of foods prepared in the home and to pilot test the adapted version using a nationally representative sample of foods. The ultimate goal of this study is to foster collaboration among scientists and policy makers to develop a food processing classification framework with greater usability to help inform nutrition communications and guidance among vulnerable populations. To our knowledge, this is the first study to consider the potential impact of categorizing home-processed foods as distinct from industrially processed foods, as well as the first study to propose an alternate classification method for home-processed foods.

## 2. Materials and Methods

The UNC system [13] was used to code foods and beverages (referred to going forward as foods) from 100 randomly selected National Health and Nutrition Examination Survey (NHANES) 24-hour dietary recalls (2015–2016; day 1 interviews) [21]. Food data included label (e.g., egg), cooking method (e.g., scrambled, fat added in cooking), and ingredients (e.g., with cheese and vegetables). All foods were independently coded by two authors (R.B-S. and C.B.). Discrepancies (*n* = 52) were reviewed and reconciled.

**Figure 1:**
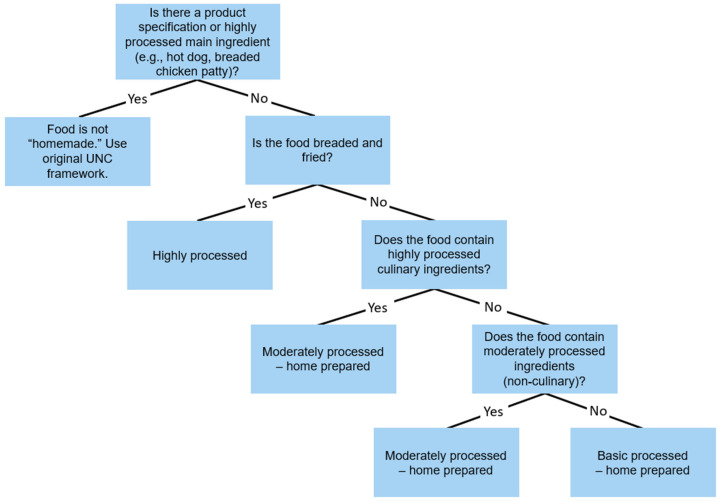
Decision tree for the home-prepared (HP) classification framework, adapted from the University of North Carolina (UNC) at Chapel Hill system [13] to include a designation between home-prepared and industrially produced foods.

The same foods were next coded using a modified version of the UNC system which incorporated a designation between home-prepared and industrially prepared mixed dishes (referred to going forward as the HP adaptation; see Table 2). This adapted framework was developed by the authors to delineate industrially processed from home-prepared foods as the items appeared in 24-hour recall data. A sample of foods drawn from NHANES recalls was used to test the HP adaptation. The final adaptation followed several rules (Figure 1). 

Rule 1: Mixed dishes and prepared foods were assumed to be home-prepared unless a product specification (e.g., frozen, ready-to-eat) or restaurant designation (e.g., Dunkin’ Donuts, McDonald’s) was provided. This contrasts the UNC framework, which assumes foods to be packaged unless specified otherwise. Prepared foods were defined as unprocessed foods requiring cooking before they can be eaten such as meats, eggs, and dried beans. Mixed dishes that contained identifiable pre-processed main ingredients (e.g., hot dog, breaded chicken patty) were not classified as homemade foods. Adding water to a pre-processed product or baking mix was not considered homemade. Foods provided by schools or cafeterias were not considered homemade. 

Rule 2: Fried foods specified as ‘breaded’, ‘battered’, or ‘coated’ (e.g. ‘Beef steak, battered, fried, lean only eaten’ (*n* = 1) and ‘Chicken thigh, fried, coated, prepared skinless, coating eaten, from raw’ (*n* = 1)) were coded as highly processed since this preparation method involves dipping the food in a refined breading or batter before deep frying in oil. Homemade items specified as ‘fried’ that did not specify a coating (e.g. ‘fried egg’ (*n* = 3) ‘fried plantain’ (*n* = 1)) were coded as basic home-prepared or moderately home-prepared depending on whether additional ingredients were included (see rules 3–5). Similarly, baked goods, chips, and sauces/dressings were coded as highly processed, unless specified as homemade.

Rule 3: A homemade food could be categorized as either basic or moderately processed in the adapted HP coding scheme. The term highly processed (i.e., ultra-processed) was reserved for industrially produced food products since they are defined as ‘multi-ingredient industrially formulated mixtures’ according the NOVA and UNC systems.

Rule 4: Home-prepared foods were classified as moderately home-prepared if they contained one or more moderately processed ingredients and/or highly processed culinary ingredients. (Culinary ingredients included any food categorized as an ingredient by the UNC system [e.g., oil: basic-ingredient; ketchup: highly-ingredient].).

Rule 5: Foods were classified as basic-home-prepared if they contained one or more basic or unprocessed/minimally processed ingredients only. If highly processed culinary ingredients were used for flavor or texture, the food was classified as moderately-home-prepared (see rule 4).

Descriptive statistics were generated using Stata Statistical Software (College Station, TX: Stata Corp LP). Percent change from UNC to HP for each processing category was calculated using Microsoft Excel, version 16.23.

## 3. Results

Demographics of the study participants whose recalls were used in the food processing classification analyses are presented in Table 3. The 100 dietary recalls included 1376 foods and 651 unique foods. Using the original UNC coding framework, there were 427 unprocessed/minimally processed foods, 113 basic processed foods, 196 moderately processed foods, and 640 highly processed foods. Using the HP adapted system, there were 377 unprocessed/minimally processed foods, 245 basic foods, 228 moderately processed foods, and 526 highly processed foods. Compared to the UNC system, foods coded as unprocessed/minimally declined by 11.7% with the HP adaptation (UNC: 31.0% vs. HP: 27.4%; basic processed foods increased by 116.8% (UNC: 8.2% vs. HP: 17.8%); moderately processed foods increased by 16.3% (UNC: 14.2% vs. HP: 16.6%) and highly processed foods decreased by 17.8% (UNC: 46.5% vs. HP: 38.2%). Several food groups exhibited frequent variability between coding schemes (Table 4). Coding discrepancies most often occurred for mixed dishes, although meat and fish, and fruits and vegetables also exhibited variability.

## 4. Discussion

The results of this analysis indicate that using explicit rules for categorizing home-prepared foods impacts processing categorization using the UNC framework, a system identified as having high inter-rater reliability relative to other frameworks [14,15]. Specifically, the basic processed category exhibited more than a 100% increase in number of foods when using an adapted framework that considers home processing. The net effect of the coding changes was an overall decrease in the number of foods categorized as highly processed within the sample population. This indicates that some homemade foods are likely to be erroneously categorized as highly processed. Such systematic misclassification by the current frameworks in use may lead to misrepresentation of the nutritional profiles of highly processed foods as more nutritious and an attenuation of the health outcomes associated with highly processed food consumption.

The above findings support previous arguments that inconsistencies in the definitions and example foods presented within and among coding schemes contributes to a lack of clarity on the issue of processed food consumption and associated health outcomes [15,19]. There is extensive epidemiological evidence that implicates excess intake of saturated fat, added sugar, and sodium as the underlying cause of many nutrition-related diseases; based on this evidence, some researchers have questioned whether processing category is just a proxy for these nutrients [19,22]. In contrast, other research suggests that highly processed food consumption may be linked to unfavorable health outcomes independent of the food’s nutritional profile [23]. For example, there is some evidence that highly (i.e., ultra-processed) foods may facilitate a loss of connection with food preparation knowledge and practice. Research indicates that highly processed food consumption is linked with less time spent preparing meals, as well as decreased confidence in one’s ability to prepare recipes [24]. A separate study found that adults who spend less time on in-home food preparation consume fast food more frequently, consume fewer fruits and vegetables, and have lower overall dietary quality than those who often prepare meals at home [25]. Future research is needed to clarify the associations between processed foods consumption, preparing foods and home vs. eating out, and dietary quality.

It is important to clarify that processing can have both positive and negative effects on a food’s nutrient profile, with optimal processing (i.e., an amount or procedure which yields maximum nutritive value) varying for each food. For example, cooking raw beans makes an inedible—even toxic—plant product into a highly nutritious food, while cooking beans in animal fat and/or adding highly processed flavoring products may compromise overall nutritive value. Such processes are often performed in the home. In addition, food products processed in the home are not necessarily more nutritious than industrially processed products; ingredients such as refined flour, sugar, or excess salt may be used in home-prepared items, and omitted in industrially prepared products. Thus, development of consumer-facing processed food frameworks, which aim to educate and provide guidance, can be leveraged to help individuals make healthful decisions in the kitchen. More research is needed to explore the efficacy of such initiatives.

While the use of processing classification systems in scientific studies has increased dramatically, non-scientific audiences’ knowledge and perceptions of these systems, and what they communicate about dietary quality, is largely unknown. The term ‘processed’ is often portrayed as synonymous with ‘junk food’ not only by some health professionals, but also by advocacy organizations and the media, and this may encourage a simplistic view of processing classification [26]. In South America, the concept of food processing has been adopted by the Pan American Health Organization to encourage consumers to avoid industrially produced foods and to combat the rapid rise in obesity [27]. Since 2014, The Dietary Guidelines for the Brazilian Population has incorporated processed food guidance into its national report, underlining that ‘ultra-processed’ foods, such as packaged snacks and soft drinks, should be avoided [28]. The Pan American Health Organization published a 2016 report that outlines a nutrient profiling model for processed and ultra-processed products [27]. This model was developed to guide norms and regulations regarding the marketing, front-of-package labeling, and fiscal policies related to processed food sales and distribution in the Americas. However, evidence of the effects of communication and policy efforts aimed at reducing processed food consumption are limited. In addition, more research is needed to develop educational approaches to teach the concept of processing to a variety of consumer audiences.

The present study demonstrates that processing classification systems may be more useful and accurate if they consider home-prepared foods as categorically distinct from industrially processed food products. However, it is important to note that home-prepared foods are not necessarily more nutritious than industrially processed foods. Home-prepared foods may still be breaded and fried, and specific nutrients to discourage (e.g., saturated fat, added sugar, and sodium) can be used in excess amounts [29]. If food processing classification schemes are to be leveraged for consumer education, this point should not be overlooked. Exploratory research indicates that consumers do consider food processing level to be associated with health and may respond to educational materials that utilize language about food processing [30,31]. Consumer-facing processing categorization systems can emphasize the dichotomy between home-prepared foods and industrially processed products, while maintaining nuanced messaging related to in-home food processing techniques and optimal processing level.

This study has several notable limitations. First, we chose to use foods gathered from 24-hour recall data from NHANES for this pilot study. Although 24-hour recalls are considered to be the ‘gold standard’ for collection of consumption data using recall methods, there are important limitations to consider within the context of this study. The purpose of conducting 24-hour recalls is generally to estimate the nutritional quality or healthfulness of diets consumed by the study population. Until recently, the processing level of diets was not explicitly considered. Information such as brand name, place of processing, added ingredients, and packaging type would be useful to assist researchers in conducting processing level analyses for future studies. Second, our sample, although randomly chosen from NHANES participants, may not be representative of the U.S. population. However, NHANES is a nationally-representative survey and it is likely that the foods consumed by individuals in our sample are typical of the larger population. Finally, the HP adaptation may not be precise enough to fully capture differences between industrially and home-prepared foods. Our decision tree for categorizing home-prepared foods does not consider factors such as food safety, scale, or the full breadth of processing techniques that may be carried out in a home setting. However, this study aimed to pilot test an alternative methodology for coding foods according to food processing category, and therefore may serve as a first step to more rigorously defining food processing categories.

## 5. Conclusions

Highly processed foods are, and will likely remain, a significant portion of the diets of individuals in the U.S. In this study, a novel HP adaptation of the UNC food processing classification framework was used to illustrate how place of processing can influence variability in the processing level of certain foods, particularly mixed dishes. The ultimate goal of this work is to encourage collaboration among scientists and policy makers to refine a food processing classification framework that can inform public health nutrition communications, help consumers make healthful dietary choices, and reduce chronic disease risk at the population level. Future research should evaluate consumer understanding of processing categorization as well as the impact of processing guidance on food choices.

## Figures and Tables

**Table 1:** Category definitions and criteria for classifying foods and beverages based on degree of industrial food processing according to the Nova [12] and UNC ^a^ [13] systems

	NOVA	Examples	UNC	Examples
Unprocessed and minimally processed	Unprocessed and minimally processed: Foods of plant origin or animal origin, shortly after harvesting, gathering, slaughter or husbanding; foods altered in ways that do not add or introduce any substance	Fresh or frozen vegetables and fruits; grains including all types of rice; freshly prepared or pasteurized non-reconstituted fruit juices; fresh, dried, frozen meats; dried, fresh, pasteurized milk.	Unprocessed and minimally processed: Single-ingredient foods with no or very slight modifications that do not change inherent properties of the food as found in its natural form.	Examples: Plain milk; fresh, frozen or dried plain fruit or vegetables; eggs, unseasoned meat; whole grain flour and pasta; brown rice; honey, herbs and spices.
Basic processed	Processed culinary ingredients: Food products extracted and purified by industry from constituents of foods, or else obtained from nature, such as salt.	Plant oils; animal fats; sugars and syrups; starches and flours, uncooked ‘raw’ pastas made from flour and water, salt.	Processed basic ingredients: single isolated food components obtained by extraction or purification using physical or chemical processes that change inherent properties of the food.	Unsweetened fruit juice not from concentrate; whole grain pasta; oil, unsalted butter, sugar, salt.
			Processed for basic preservation or precooking: single minimally processed foods modified by physical or chemical processes for the purpose of preservation or precooking but remaining as single foods.	Unsweetened fruit juice from concentrate; unsweetened/unflavored canned fruit, vegetables, legumes; plain peanut butter, refined grain pasta, white rice; plain yogurt.
Moderately processed	Processed foods: Manufactured by adding substances like oil, sugar or salt to whole foods, to make them durable and more palatable and attractive.	Canned or bottled vegetables in brine; fruits preserved in syrup; tinned whole or pieces of fish preserved in oil; salted nuts; un-reconstituted processed meat and fish such as ham, bacon, smoked fish; cheese.	Moderately processed for flavor: single minimally or moderately processed foods with addition of flavor additives for the purpose of enhancing flavor	Sweetened fruit juice, flavored milk; frozen french fries; salted peanut butter; smoked or cure meats; cheese, flavored yogurt, salted butter.
			Moderately processed grain products: grain products made from whole-grain flour with water, salt, and/or yeast.	Whole grain breads, tortillas or crackers with no added sugar or fat.
Highly processed	Ultra-processed foods: Formulated mostly or entirely from substances derived from foods. Processes include hydrogenation, hydrolysis; extruding, molding, reshaping; pre-processing by frying, baking.	Confectionery; burgers and hot dogs; breaded meats; breads, buns, cookies (biscuits); breakfast cereals; ‘energy’ bars; sauces; cola, ‘energy’ drinks; sweetened yoghurts; fruit and fruit ‘nectar’ drinks; pre-prepared dishes.	Highly processed ingredients: multi-ingredient industrially formulated mixtures processed to the extent that they are no longer recognizable as their original plant/animal source.	Tomato sauce, salsa, mayonnaise, salad dressing, ketchup.
			Highly processed stand-alone: multi-ingredient industrially formulated mixtures processed to the extent that they are no longer recognizable as their original plant/animal source.	Soda, fruit drinks; formed lunchmeats; breads made with refined flours; pastries; ice-cream, processed cheese; candy; pre-packaged and prepared vegetable, legume, meat and fish-based dishes.

^a^ University of North Carolina at Chapel Hill.

**Table 2:** The ‘home processing’ food processing classification system adapted from the University of North Carolina at Chapel Hill^13^ framework to differentiate between home-prepared and industrially produced mixed dishes (adapted categories and definition shown in italics).

	Examples
Unprocessed and minimally processed foods	
Basic processed foods	
Basic-ingredient	
Basic-preservation	
Basic home-prepared: Foods containing one or more processed basic ingredients or foods processed for basic preservation or precooking.	Chicken breast (home-cooked) ^a^; fried egg ^b^; mashed potatoes made with milk ^b^; homemade salsa ^c^
Moderately processed foods	
Moderately-flavor	
Moderately-grain product	
Moderately home-prepared: Food containing one or more moderately processed ingredients and/or highly processed culinary ingredients	Burrito with meat and beans ^c^; meat loaf ^c^; tuna salad ^c^; pasta with tomato-based sauce, meat and/or vegetables ^c^
Highly processed foods	
Highly-ingredient	
Highly stand-alone	

^a^ Foods coded as ‘minimally processed’ using the UNC framework. ^b^ Foods coded as ‘moderately processed’ using the UNC framework. ^c^ Foods coded as ‘highly processed’ using the UNC framework.

**Table 3:** Baseline demographic characteristics, 100 respondents with day 1 dietary recalls randomly selected from the National Health and Examination Survey 2015–2016

	Sample (*n* = 100)
**Sex, % female**	56
**Age, (years) mean (sd); range (years)**	31 (24); 2–80
Race/ethnicity, %	
Non-Hispanic white	34
Non-Hispanic black	23
Mexican American	11
Hispanic	18
Multiracial/Asian/American Indian/other	14
Education, % ^a^	
High school degree or less	17
High school/GED or equivalent	28
Some college or associate’s degree	37
College graduate or above	19
Annual household income, %	
<$20,000	1
$20,000–$44,999	26
$45,000–$74,999	31
$75,000–$99,999	9
>$100,000	10

^a^ Adults 20 years and older, *n* = 54.

**Table 4:** Frequency of coding discrepancies by food category between the University of North Carolina at Chapel Hill (UNC) 21 and adapted home processing (HP) frameworks for foods from 100 randomly selected dietary recalls, NHANES 2015–2016 (N = 1,376)

Category	UNC (N)	HP	Frequency	% Change	Example Foods
Meat and Fish	Highly (640)	Moderately	4	0.63	Chicken breast, grilled with sauce
					Beef with Barbeque sauce
	Unprocessed/minimally (427)	Basic	32	7.49	Pork, NS ^a^ as to cut, cooked, NS as to fat eaten
					Chicken wing, baked, broiled, or roasted from raw
	Moderately (196)	Basic	2	1.02	Tilapia, baked or broiled with oil
Mixed dishes (rice, grain or vegetable based)	Highly (640)	Basic	23	3.59	Rice with beans and tomatoes
					Stuffed pepper, with rice and meat
					Rice, white, with peas and carrots, NS as to fat added in cooking
	Highly (640)	Moderately	37	5.78	Burrito with meat and beans
					Meat loaf made with beef
					Pasta with tomato-based sauce, meat, and added vegetables, home recipe
Grains and grain-based products	Unprocessed/minimally (427)	Basic	1	0.23	Barley, fat not added in cooking
	Highly (640)	Moderately	6	0.94	Cornbread, made from home recipe
Egg-based dishes	Moderately (196)	Basic	7	3.57	Egg, whole, fried with oil
	Highly (640)	Basic	4	0.63	Egg omelet or scrambled egg, with potatoes and/or onions, fat added in cooking
	Highly (640)	Moderately	3	0.47	Egg omelet or scrambled egg, with cheese and vegetables, fat added in cooking
Soups	Highly (640)	Basic	10	1.56	Chicken or turkey vegetable soup, home recipe
Vegetables	Unprocessed/minimally (427)	Basic	19	4.45	Broccoli, cooked, from fresh, fat not added in cooking
					Vegetable combinations, Asian style, cooked, fat not added in cooking
	Moderately (196)	Basic	17	8.67	Collards, cooked, from fresh, made with oil
					Potato, mashed, from fresh, made with milk
Sauces, spreads, and dressings	Highly (640)	Basic	8	1.25	Guacamole
					Salsa, red, homemade
	Highly (640)	Moderately	5	0.78	Gravy, beef or meat
Drinks	Moderately (196)	Basic	3	1.53	Fruit smoothie, with whole fruit and dairy
TOTAL			181	13.15	

^a^ Not specified.
